# The CHEK2^*^1100delC mutation has no major contribution in oesophageal carcinogenesis

**DOI:** 10.1038/sj.bjc.6601551

**Published:** 2004-02-17

**Authors:** L B Koppert, M Schutte, M Abbou, H W Tilanus, W N M Dinjens

**Affiliations:** 1 Department of Pathology, Erasmus university Medical Center, Josephine Nefkens Institute, PO Box 1738, Rotterdam 3000 DR, The Netherlands; 2 Department of Surgery, Erasmus university Medical Center, PO Box 1738, Rotterdam 3000 DR, The Netherlands; 3 Department of Medical Oncology, Erasmus university Medical Center, Josephine Nefkens Institute, PO Box 1738, Rotterdam 3000 DR, The Netherlands

**Keywords:** *CHEK2*, *CHK2*, mutation analysis, oesophageal cancer, Barrett's oesophagus

## Abstract

In response to DNA damage, the cell cycle checkpoint kinase 2 (CHEK2) may phosphorylate p53, Cdc25A and Cdc25C, and regulate BRCA1 function, leading to cell cycle arrest and DNA repair. The truncating germline mutation CHEK2^*^1100delC abrogates kinase activity and confers low-penetrance susceptibility to breast cancer. We found CHEK2^*^1100delC in 0.5% of 190 oesophageal squamous cell carcinomas and in 1.5% of 196 oesophageal adenocarcinomas. In addition, we observed the mutation in 3.0% of 99 Barrett's metaplasias and 1.5% of 66 dysplastic Barrett's epithelia, both known precursor lesions of oesophageal adenocarcinoma. Since CHEK2^*^1100delC mutation frequencies did not significantly differ among oesophageal squamous cell carcinomas, adenocarcinomas and (dysplastic) Barrett's epithelia, as compared to healthy individuals, we conclude that the CHEK2^*^1100delC mutation has no major contribution in oesophageal carcinogenesis.

Oesophageal carcinoma is the ninth most common tumour type worldwide. Despite surgical intervention, 5-year overall survival is less than 20%, mainly due to the fact that patients often present with an advanced tumour stage. Alcohol and tobacco use are established risk factors for the development of oesophageal squamous cell carcinomas (Muir and McKinney, 1992). The presence of Barrett's oesophagus is the main risk factor for adenocarcinoma formation, being 30–125 times higher in patients with Barrett's oesophagus, as compared to the general population. Barrett's oesophagus is defined as a columnar cell metaplasia of the native distal oesophageal squamous cell epithelium (Spechler and Goyal, 1986), accompanied by the presence of Goblet cells, as a result of chronic gastro-oesophageal reflux. Barrett's metaplasia can progress to low- and high-grade dysplasia, and ultimately to invasive and metastasising adenocarcinoma. Patients with Barrett's oesophagus receive endoscopic surveillance to detect dysplasia and to diagnose carcinoma at an early and possibly treatable stage. The identification of genes that confer susceptibility for adenocarcinoma formation in Barrett's oesophagus would imply improved manageability of patients with Barrett's oesophagus. Familial cases of oesophageal cancer are however rare, and susceptibility genes for oesophageal cancer are thus unlikely to be found by linkage analysis. Consequently, screening of candidate susceptibility genes may be a more feasible approach for oesophageal cancer.

*CHEK2* (also known as *CHK2*) is the mammalian homologue of *Saccharomyces cerevisiae* Rad53 and *Schizosaccharomyces pombe* Cds1 genes (Matsuoka *et al*, 1998; Chaturvedi *et al*, 1999). The *CHEK2* gene, located on human chromosome 22q12, encodes a cell cycle checkpoint kinase that is implicated in DNA damage responses. Phosphorylation of the p53, Cdc25A and Cdc25C protein results in arrests in various phases of the cell cycle (Zhou and Elledge, 2000; Bartek *et al*, 2001). In addition, *CHEK2* has been implicated in the regulation of DNA repair by the BRCA1 protein (Zhou and Elledge, 2000; Bartek *et al*, 2001). CHEK2^*^1100delC is a truncating germline variant of *CHEK2* that abrogates kinase activity (Lee *et al*, 2001; Wu *et al*, 2001) and has initially been reported in families suffering from the Li–Fraumeni syndrome without *p53* mutations (Bell *et al*, 1999). In familial gastric cancers, which are known to cluster in Li–Fraumeni families, germline *CHEK2* mutations were absent (Kimura *et al*, 2000). In sporadic (osteo)sarcomas, lung cancers, breast cancers, ovarian cancers, colon cancers and haematopoietic neoplasms, *CHEK2* was found to be rarely mutated (Bell *et al*, 1999; Haruki *et al*, 2000; Hofmann *et al*, 2001; Tavor *et al*, 2001; Aktas *et al*, 2002; Hangaishi *et al*, 2002; Ingvarsson *et al*, 2002; Miller *et al*, 2002). From recent publications, it appeared that the germline CHEK2^*^1100delC mutation in fact confers low-penetrance susceptibility to breast cancer (Meijers-Heijboer *et al*, 2002; Vahteristo *et al*, 2002). An increased frequency of CHEK2^*^1100delC was found among breast carcinoma families without *BRCA1* or *BRCA2* mutations, associated with an approximately two-fold increase of breast cancer risk in female carriers (Meijers-Heijboer *et al*, 2002).

The p53 protein is one of the downstream targets of CHEK2 kinase. Mutations of the *p53* gene result in a variety of disturbances in growth control involving DNA replication, DNA repair and apoptosis. Like in breast carcinoma, mutations of the *p53* gene appear to play an important role in the development of oesophageal squamous cell carcinoma, dysplastic Barrett's epithelium and the progression to oesophageal adenocarcinoma (Casson *et al*, 1991; Neshat *et al*, 1994; Wu *et al*, 1998). Ample studies have reported mutations in *p53* in oesophageal carcinomas, with mutation frequencies varying from 40 to 90% (Jankowski *et al*, 1999; Mandard *et al*, 2000; Wijnhoven *et al*, 2001; Jenkins *et al*, 2002). As *CHEK2* and *p53* are thought to be participants of the same biological pathway, we aimed to establish whether CHEK2^*^1100delC confers susceptibility to oesophageal cancer, by determining the frequency of the mutation among an unselected series of oesophageal cancers and precursor lesions.

## MATERIALS AND METHODS

### Tissue specimens and controls

We investigated a cohort of 190 oesophageal squamous cell cancer patients, 196 oesophageal adenocarcinoma patients, 99 patients with Barrett's metaplasia and 66 patients with dysplastic Barrett's epithelium. Tissue samples were obtained from resection specimens (carcinomas) or endoscopic biopsies (Barrett's metaplasia and dysplasia), all derived from different patients. We microscopically confirmed that the endoscopic biopsy specimens did not exhibit any tumour cell invasion. Tissue fragments were digested from routine formalin-fixed, paraffin-embedded tissue blocks, without deparaffinisation, in 180 *μ*l of 50mM l^−1^ Tris/HCl (pH=8.0), and 20 *μ*l of Proteinase K (20 mg *μ*l^−1^) was added. After overnight incubation at 56°C, the lysates were boiled for 10 min and subsequently centrifuged. The two series of Dutch control individuals consisted of: (A) 184 spouses of individuals heterozygous with respect to cystic fibrosis from the Southwest Netherlands, and (B) 460 individuals at ages 55 and older, ascertained through the Erasmus Rotterdam Health and the Elderly Study (ERGO) (Meijers-Heijboer *et al*, 2002).

## METHODS

### Allele-specific oligonucleotide hybridisation assay

*CHEK2* exon 10 was amplified using forward *CHEK2* primer (5′-CAACATTATTCCCTTTTGTACTG-3′) and reverse *CHEK2* primer (5′-GTTCCACATAAGGTTCTCATG-3′). DNA samples (1 *μ*l) were subjected to PCR analysis in a total volume of 50 *μ*l containing 1 × Promega buffer, 1.5 mM MgCl_2_-solution, 4 *μ*M dGTP, dTTP, dCTP and dATP, 3 U Taq DNA polymerase (Promega, Madison, USA), and 0.2 *μ*g of forward and reverse *CHEK2* primers. PCR amplification consisted of 35 cycles (95°C for 30 s, 55°C for 45 s and 72°C for 45 s), followed by a final extension at 72°C for 10 min. We detected the CHEK2^*^1100delC mutation by application of diluted PCR products to nylon filters and hybridisation under a high stringency of [^32^P]-labelled oligonucleotides complementary to CHEK2^*^1100delC and the wild-type sequence (5′-TTAGATTATGATTTTGGG-3′ and 5′-TTAGATTACTGATTTTGG-3′, respectively).

### Polymorphic marker analysis

DNA was radioactively amplified essentially, as described above, using forward primer (5′-TAAGGTGGGAGGTTCACTTG-3′) and reverse primer (5′-ACCCATCCTCCTGCCTTAG-3′) for the D22S275 locus. PCR products were separated on a 6% polyacrylamide denaturing gel. After electrophoresis, gels were dried on blotting paper and exposed to X-ray films. Films were evaluated by visual inspection.

### Immunohistochemistry

From formalin-fixed paraffin-embedded tissue blocks, 4-*μ*m thick sections were mounted on 3-aminopropyl-triethoxysilane (APES)-coated glass slides. The sections were incubated with a mouse monoclonal antibody DCS 270.1 against the human CHEK2 protein (Novocastra Laboratories, Newcastle, UK; at a dilution of 1 : 50). Immunoreactivity was visualised by a standard avidin biotin immunoperoxidase technique, using a commercially available kit (Labvision, Fremont, USA) (Bartkova *et al*, 2001; Lukas *et al*, 2001).

### Statistics

Differences of the CHEK2^*^1100delC mutation frequency between patients and controls were expressed in terms of odds ratios (OR) and 95% confidence intervals (95% CI), and tested with the *χ*^2^-test.

## RESULTS AND DISCUSSION

We analysed tumour and biopsy samples obtained from 551 Dutch patients by a CHEK2^*^1100delC allele-specific oligonucleotide hybridisation assay. CHEK2^*^1100delC mutations were detected in 0.5% of 190 squamous cell carcinomas, 1.5% of 196 adenocarcinomas, 3.0% of 99 Barrett's metaplasias and in 1.5% of 66 dysplasias (Table 1

**Table 1:** CHEK2^*^1100delC mutation frequencies

CHEK2^*^1100delC mutation					
	**Number tested**	**Carriers**	**Percentage**	**OR (95% CI)**^a^	***P*-value^a^**
Controls					
Netherlands (A)^b^	184	3	1.6		
Netherlands (B-ERGO)^b^	460	6	1.3		
Total	644	9	1.4		
Barrett's metaplasias	99	3	3.0	2.20 (0.38–9.04)	0.23
Dysplasias	66	1	1.5	1.09 (0.02–8.05)	0.93
Adenocarcinomas	196	3	1.5	1.10 (0.19–4.45)	0.89
Squamous cell carcinomas	190	1	0.5	0.37 (0.01–2.73)	0.33
Total	551	8	1.5	1.04 (0.35–3.06)	0.94

a OR: odds ratio, 95% CI: 95% confidence interval, and *P*-values are determined by *χ*^2^-test, as compared to frequency in controls.

b CHEK2^*^1100delC frequency in Dutch control cohorts A and B by Meijers-Heijboer *et al* (2002).

). *χ*^2^ analysis revealed no significant differences between patient groups and the CHEK2^*^1100delC mutation frequency of 1.4% among 644 control individuals (*P*=0.94), and the odds ratio of the total patient group compared to the controls was 1.04 (95% CI 0.35–3.06, Table 1). These results suggest that CHEK2^*^1100delC does not substantially contribute to the development of oesophageal carcinoma. CHEK2^*^1100delC could still confer a three-fold risk, which is greater than the estimated two-fold risk associated with breast cancer, and CHEK2^*^1100delC thus may still be a low-penetrance susceptibility gene to oesophageal cancer. Given the low frequency of the mutation, however, even the maximal possible three-fold risk conferred by CHEK2^*^1100delC would only marginally contribute to the overall incidence of oesophageal cancer.

**Figure 1:**
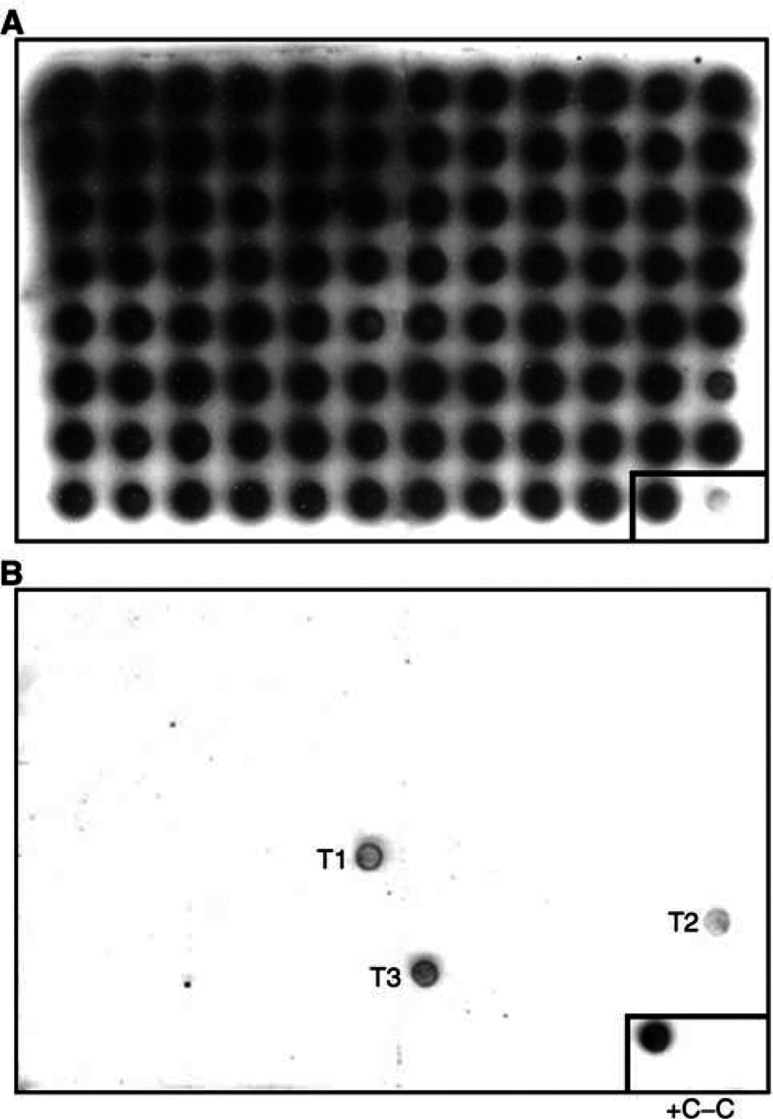
Allele-specific oligonucleotide hybridisation assay of 94 adenocarcinomas. Blot (**A**), hybridisation with wild-type oligonucleotide. Blot (**B**), hybridisation with mutant oligonucleotide. Adenocarcinoma samples T1, T2 and T3 are positive for the CHEK2^*^1100delC mutation, sample ‘+C’ represents a control individual with CHEK2^*^1100delC, and sample ‘−C’ represents a control individual negative for CHEK2^*^1100delC.

Examples of the hybridisation assay are shown in Figure 1

. The median ages of patient groups at diagnosis were 59.6, range 14–86 years (Barrett's metaplasias), 60.3, range 32–84 years (dysplasias), 63.9, range 36–84 years (adenocarcinomas) and 60.9, range 31–79 years (squamous cell carcinomas). Ages at diagnosis of CHEK2^*^1100delC mutation carriers were 48, 73 and 77 years (Barrett's metaplasias), 59 years (dysplasia), 44, 50 and 63 years (adenocarcinomas) and 73 years (squamous cell carcinoma), which were not different from noncarriers and, again, not supporting a major role of CHEK2^*^1100delC in oesophageal cancer predisposition.

**Figure 2:**
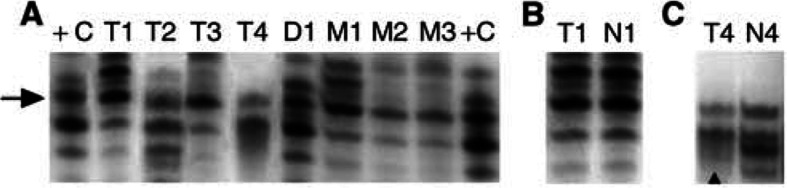
D22S275 polymorphic marker analysis in samples with CHEK2^*^1100delC. (**A**), the allele (arrow), known to be present in all carriers of CHEK2^*^1100delC (18), is present in all samples with the CHEK2^*^1100delC mutation (T1–T3 mutated adenocarcinoma samples, T4 mutated squamous cell carcinoma, D1 mutated dysplastic tissue, M1–M3 mutated metaplastic tissue), ‘+C’ represents a control individual with CHEK2^*^1100delC. (**B**, **C**), LOH patterns from two mutated tumours (T1 adenocarcinoma and T4 squamous cell carcinoma) compared with the corresponding normal tissues (N1 and N4) are shown. The arrowhead points to the deleted allele in T4.

All mutations were confirmed and proven to be germline-derived by investigating patients' normal tissues. Only paraffin-embedded samples of tumour-negative lymph nodes were available, precluding confirmation of mutations by sequencing of long-range PCR products (Sodha *et al*, 2002). The CHEK2^*^1100delC germline mutation has, however, been found to be linked to one specific allele of the D22S275 polymorphic marker, that is located in intron 4 of *CHEK2*, which is present in 13% of the Dutch population (Meijers-Heijboer *et al*, 2002). All eight mutation-positive cases were demonstrated to carry the D22S275 allele linked to the *CHEK2* mutation, which supports the detected mutations (Figure 2

). Comparison of allele patterns in mutated tumours with their normal tissues revealed LOH in only one of the three informative carcinomas (T1 without LOH in Figure 2B, T4 with LOH in Figure 2C). LOH was also observed in three out of 14 informative nonmutated tumour samples. Limited data are, however, available on LOH of *CHEK2* in tumours, and the possible tumour-suppressing role of *CHEK2* therefore awaits further studies.

**Figure 3:**
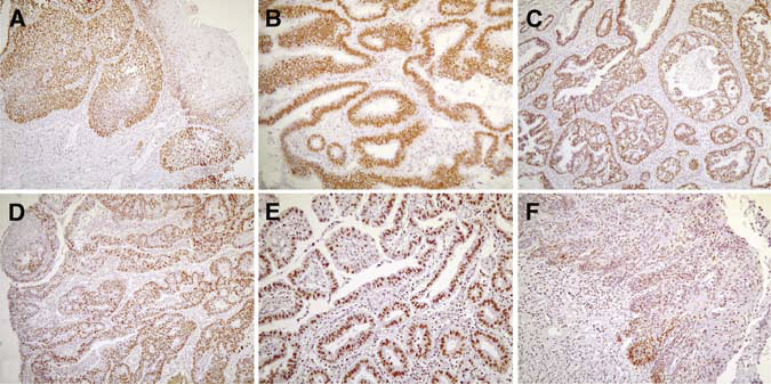
CHEK2 protein expression. Nonmutated squamous cell carcinoma in (**A**), nonmutated adenocarcinoma in (**B**). The remaining samples are from the four mutated tumours: adenocarcinomas T1, T2 and T3 shown in (**C**–**E**), squamous cell carcinoma T4 shown in (**F**). Magnification × 100 (**B**, **E**), × 50 (**A**, **C**, **D**, **F**). Note the strong nuclear CHEK2 immunoreactivity in the tumour cells.

Immunohistochemistry using monoclonal antibody DCS 270.1 on a series of mutated and nonmutated tumour tissues showed clear nuclear staining in all cases (Figure 3

). Since the DCS 270.1 epitope lies within the N-terminus of CHEK2, staining of both wild-type and mutant protein may be expected. We observed no differences in CHEK2 protein levels, that is, neither lower intensity nor a lower percentage of CHEK2-positive cells, between CHEK2^*^1100delC mutated and nonmutated cancers. This was also true for the single-mutated tumour with LOH (T4 in Figure 3f), suggesting that a theoretical two-fold reduction in CHEK2 protein level cannot be detected by the applied immunohistochemistry method. This appears to contrast the results of Vahteristo *et al*, who reported loss of expression in three of the four CHEK2^*^1100delC tumours and reduction of CHEK2 protein expression in the fourth, using the same antibody (Vahteristo *et al*, 2002). Comparison of the two studies is, however, difficult, as they did not indicate the precise level of reduction in protein expression (Vahteristo *et al*, 2002). Since only few CHEK2^*^1100delC tumours have currently been reported, both the data of Vahteristo *et al* and the present data should be interpreted with caution.

In summary, our study of a large and unselected series of Barrett's metaplasias and dysplasias, oesophageal adenocarcinomas and squamous cell carcinomas suggests that the germline CHEK2^*^1100delC mutation has no major contribution in oesophageal carcinogenesis.
